# Identification of *Rothia* Bacteria as Gluten-Degrading Natural Colonizers of the Upper Gastro-Intestinal Tract

**DOI:** 10.1371/journal.pone.0024455

**Published:** 2011-09-21

**Authors:** Maram Zamakhchari, Guoxian Wei, Floyd Dewhirst, Jaeseop Lee, Detlef Schuppan, Frank G. Oppenheim, Eva J. Helmerhorst

**Affiliations:** 1 Department of Periodontology and Oral Biology, Boston University Henry M. Goldman School of Dental Medicine, Boston, Massachusetts, United States of America; 2 Department of Molecular Genetics, Forsyth Institute, Cambridge, Massachusetts, United States of America; 3 Division of Gastroenterology, Beth Israel Deaconess Medical Center, Harvard Medical School, Boston, Massachusetts, United States of America; 4 Division of Molecular and Translational Medicine, Johannes-Gutenberg-University, Mainz, Germany; East Carolina University School of Medicine, United States of America

## Abstract

**Background:**

Gluten proteins, prominent constituents of barley, wheat and rye, cause celiac disease in genetically predisposed subjects. Gluten is notoriously difficult to digest by mammalian proteolytic enzymes and the protease-resistant domains contain multiple immunogenic epitopes. The aim of this study was to identify novel sources of gluten-digesting microbial enzymes from the upper gastro-intestinal tract with the potential to neutralize gluten epitopes.

**Methodology/Principal Findings:**

Oral microorganisms with gluten-degrading capacity were obtained by a selective plating strategy using gluten agar. Microbial speciations were carried out by 16S rDNA gene sequencing. Enzyme activities were assessed using gliadin-derived enzymatic substrates, gliadins in solution, gliadin zymography, and 33-mer α-gliadin and 26-mer γ-gliadin immunogenic peptides. Fragments of the gliadin peptides were separated by RP-HPLC and structurally characterized by mass spectrometry. Strains with high activity towards gluten were typed as *Rothia mucilaginosa* and *Rothia aeria*. Gliadins (250 µg/ml) added to *Rothia* cell suspensions (OD_620_ 1.2) were degraded by 50% after ∼30 min of incubation. Importantly, the 33-mer and 26-mer immunogenic peptides were also cleaved, primarily C-terminal to Xaa-Pro-Gln (XPQ) and Xaa-Pro-Tyr (XPY). The major gliadin-degrading enzymes produced by the *Rothia* strains were ∼70–75 kDa in size, and the enzyme expressed by *Rothia aeria* was active over a wide pH range (pH 3–10).

**Conclusion/Significance:**

While the human digestive enzyme system lacks the capacity to cleave immunogenic gluten, such activities are naturally present in the oral microbial enzyme repertoire. The identified bacteria may be exploited for physiologic degradation of harmful gluten peptides.

## Introduction

Celiac disease, an intestinal inflammatory disease with auto-immune features, is caused by oral ingestion of gluten peptides that escape intestinal degradation. These peptides are antigenically presented on HLA-DQ2 or HLA-DQ8, preferentially after deamidation of certain glutamines by the celiac disease auto-antigen tissue transglutaminase (tTG), eliciting a destructive Th1 T cell response [1], [2]. Gluten proteins that are found in wheat, barley and rye are abundantly present in the Western diet. Strict elimination of gluten from the diet is the therapy of choice, usually leading to a significant improvement of clinical symptoms characteristic of celiac disease related to malabsorption, fatigue and possibly associated autoimmunity, and largely restores the duodenal mucosal damage (villous atrophy, crypt hyperplasia, T cell infiltration). A strictly gluten-free diet, however, is difficult to maintain and poses a significant social and financial burden to the patient. Therefore, an additive non-dietary therapy that relieves patients from a highly restricted gluten free diet is much needed [3], [4].

A novel therapeutic approach for celiac disease is the use of enzymes to achieve proteolytic fragmentation of gluten proteins which otherwise escape proteolytic inactivation by gastric, pancreatic and intestinal brush border enzymes into smaller non-immunogenic peptides. The resistance of gluten to digestive proteases is due to a particular primary structure based on their high number of proline (P) and glutamine (Q) residues, and repetitive PQ sequences that are not cleavable by common GI proteases [5]. A 33-mer peptide present in α-gliadins [6] (also denoted as “superantigen” [7]), and a 26-mer peptide from γ-gliadins [8] are particularly resistant to degradation, while carrying multiple potent HLA-DQ2 binding T cell stimulatory epitopes after deamidation by tTG [9]. Abolishment of these and related peptides by proteolytic enzymes, e.g. provided as a dietary supplement, could conceivably reduce immunotoxicity of gluten for celiac patients [10].

A number of gluten-degrading enzymes from microbial and cereal sources have been discovered. Prolyl-endopeptidases target the conformationally constrained peptide bonds C-terminal to proline residues. Prolyl endopeptidases from *Sphingomonas capsulata*, *Flavobacterium meningosepticum*, *Myxococcus Xanthus* and *Aspergillus niger* have been pursued as drug candidates for enzymatic treatment of gluten in celiac disease [11], [12]. Another enzyme with such therapeutic potential is a glutamine endoprotease from barley designated EP-B2 [13]. Prolyl endopeptidases, alone or in combination with EP-B2, have been demonstrated to neutralize immunotoxicity of a pre-digested gluten preparation *in vitro*, *ex vivo* and *in vivo* [14], [15], [16], [17], [18]. While promising in terms of gluten-degrading activities, all enzymes referred to above originate from species not naturally associated with the human body. In addition, toxicity of *F. meningosepticum* and *A. niger* limits their usefulness as probiotic agents.

A biologically more favorable and likely source for gluten-degrading enzymes would be the microbiome colonizing the human gastro-intestinal tract. It is well recognized that bacteria populating the human body supply the host with numerous functions that are not encoded by the human genome [19]. For instance, bacteria that colonize the large intestine ferment starches that are resistant to mammalian digestive enzymes [20]. Recent work in our laboratory has shown that gluten-degrading bacteria are naturally residing in the oral cavity [21], [22]. The discovery of oral enzymes with such activities is significant, since the oral cavity represents the entrance to the digestive tract that all ingested food has to pass and in which gluten is mixed with the oral microorganisms in human saliva. The finding of gluten-degrading oral microbes may serve as a novel source for therapeutic gluten degrading enzymes. In this study we isolated and identified gluten-degrading microorganism(s) from human saliva and plaque samples and functionally characterized the enzymes with regard to their capacity to degrade immunogenic gliadin peptides.

## Materials and Methods

### Collection of dental plaque and whole saliva samples

This study, involving the collection of human dental plaque and whole saliva, was approved by the Institutional Review Board at Boston University, protocol number H-23709. Written informed consent was obtained from the participating subject prior to participation. The enrolled subject presented with good oral health without overt signs of gingival inflammation or other oral or systemic conditions. Supragingival plaque was collected from interproximal dental spaces with an explorer 24 h after refraining from oral hygiene. The plaque material was suspended in 500 µl saliva ion buffer containing 50 mM KCl, 1.5 mM potassium phosphate, 1 mM CaCl_2_ and 0.1 mM MgCl_2_, pH 7.0. Masticatory stimulated whole saliva (5 ml) was obtained by expectoration as described [23].

### Culturing of oral microorganisms

Aliquots of 50 µl of 1∶1,000 diluted dental plaque or whole saliva suspensions were plated on gluten-limited agar (GA) containing per liter: 23 g wheat gluten (Sigma-Aldrich, St. Louis, MO; catalog number G5004, >80% protein), 5 g sodium chloride (Fisher, Pittsburg, PA), 1 g soluble starch (Fisher), 12 g agar (Fluka, Sigma-Aldrich), 0.4 g sodium bicarbonate (Fisher), 1 g glucose (Sigma-Aldrich), 1 g sodium pyruvate (Sigma-Aldrich), 0.5 g cysteine (Sigma-aldrich), 0.01 g haemin and 0.001 g vitamin K (Beckton-Dickinson, Franklin Lakes, NJ), 1 g L-arginine (Sigma-Aldrich), 0.25 g soluble pyrophosphate (Fisher) and 0.5 g sodium succinate (Fisher). Incubations were carried out at 37°C under aerobic conditions or in a sealed candle jar that was rendered anaerobic using GasPak pouches (Beckton-Dickinson, Franklin Lakes, MD). Individual colonies were transferred to GA plates and after 48 incubation were subcultured on *Brucella* agar (Hardy Diagnostics, Santa Maria, CA). Subculturing on *Brucella* agar plates was repeated until cultures were obtained that were macroscopically and microscopically pure. The strains were then plated once more on GA to confirm growth on this selective agar formulation. From these slabs bacterial stocks were prepared which were kept at −80°C in a glycerol/BHI broth mixture (20/80% v/v).

### Microbial speciation by 16S rDNA

Microbial colonies with gliadin-degrading activity were identified by 16S rDNA analysis. DNA extraction was performed using the UltraClean™ Microbial DNA Isolation Kit (Mo Bio Laboratories, Carlsbad, CA) following the manufacturer's instructions for the isolation of genomic DNA from Gram-positive bacteria. Purified DNA was sequenced using an ABI prism cycle-sequencing kit (BigDye® Terminator Cycle Sequencing kit) on an ABI 3100 Genetic Analyser (Applied Biosystems, Foster City, CA). Reactions used a quarter-dye chemistry as previously described [24], [25]. Partial sequences were identified by BLASTN analysis against the Human Oral Microbiome Database (http://www.homd.org) containing sequencing of over 35,000 clones and isolates. Sequences were assembled from the ABI electropherogram files using Sequencher 4.9 (Gene Codes Corporation, Ann Arbor, MI).

### Degradation of paranitroanilide-derived substrates

The hydrolysis of four gliadin-derived substrates, benzyloxycarbonyl-YPQ-paranitroanilide (Z-YPQ-pNA), Z-QQP-pNA, Z-PPF-pNA and Z-PFP-pNA, was assessed spectrophotometrically at 405 nm as described previously [22].

### Degradation of gliadins in-solution

A mixture of gliadins was purchased from Sigma (St. Louis, MO), dissolved to 5 mg/ml in 60% (v/v) ethanol and diluted to 250 µg/ml in suspensions of bacterial strain WSA-8 (*R. aeria*; final OD_620_ 1.0). Incubation mixture aliquots (100 µl) were removed after various incubation times, dried and analyzed on pre-cast 12% gels (Novex, InVitrogen, Carlsbad, CA).

### Degradation of 33-mer and 26-mer gliadin domains

Synthetic highly immunogenic peptides derived from α2-gliadin (LQLQPFPQPQLPYPQPQLPYPQPQLPYPQPQPF; 33-mer) [6] or γ-gliadin (FLQPQQPFPQQPQQPYPQQPQQPFPQ; 26-mer) [8] were synthesized at a purity of 95% (21^st^ Century Biochemicals, Marlboro, MA), dissolved in deionized water to 10 mg/ml. The peptides (final concentration 250 µg/ml) were incubated with trypsin or chymotrypsin (1 µg/ml in 50 mM ammonium bicarbonate, pH 8.0), or pepsin (1 µg/ml in 0.1 M HCl, pH 1.0) or strain WSA-8 (*R. aeria*) in saliva ion buffer (OD_620_ 1.2, pH 7.0). Degradation of the 33-mer and 26-mer over time was monitored in 100 µl aliquots by RP-HPLC as reported previously [22].

### RP-HPLC

The 100 µl sample aliquots were mixed with 900 µl buffer A containing 0.1% (v/v) trifluoroacetic acid (TFA), filtered and analyzed by RP-HPLC using a HPLC Model 715 (Gilson, Middleton, WI) and a C-18 column (TSK-GEL 5 µm, ODS-120T, 4.6×250 mm, TOSOHaas, Montgomeryville, PA). Peptides were eluted using a linear gradient from 0% to 55% buffer B containing 80% (v/v) acetonitrile and 0.1% (v/v) TFA over a 75 min time interval at a flow rate of 1.0 ml/min. The eluate was monitored at 219 and 230 nm and eluting fractions were collected using peak width and peak sensitivity settings of 1.2 and 5, respectively (Unipoint version 3.3, Gilson). The collected peaks were dried using a speedvac (Savant, ThermoFisher Scientific, Waltham, MA).

### Mass Spectrometric Characterization of 33-mer/26-mer fragments by LC-ESI-MS/MS

All dried peaks were dissolved in 25 µl of a solution containing 5% acetonitrile and 0.1% formic acid. Mass spectrometry was conducted using a capillary nano-flow liquid chromatography and electrospray ionization tandem mass spectrometer (LC-ESI-MS/MS) as previously described [22], [26]. The raw MS/MS data of the peptide ions in each of the analyzed samples were searched against an in-house generated database containing the sequences of the 33-mer and the 26-mer using SEQUEST software (Bioworks Browser 3.3.1, Thermo-Finnigan). X-corr values applied were 1.5, 2.2 and 3.5 for Z = 1, 2, and 3, respectively. The deltaCn and peptide probability values were set at >0.1 and <0.01 which avoided false-positive identifications as reported. [22]

### Gliadin zymography

Bacterial strains WSA-2B (*R. mucilaginosa*), WSA-8 (*R. aeria*), WSA-26 (*R. mucilaginosa*) and *R. mucilaginosa* ATCC 25296 were harvested from Brucella agar and suspended in saliva ion buffer to a final OD_620_ of 5.0. A 150 µl aliquot was centrifuged, the cell pellet resuspended in 20 µl zymogram sample buffer and analyzed by gliadin zymography as described [22] except that the separating gel contained 6% instead of 8% acrylamide to achieve a better separation of proteins in the 70–75 kDa region.

## Results

### Isolation and speciation of gluten-degrading oral bacteria

**Figure 1:**
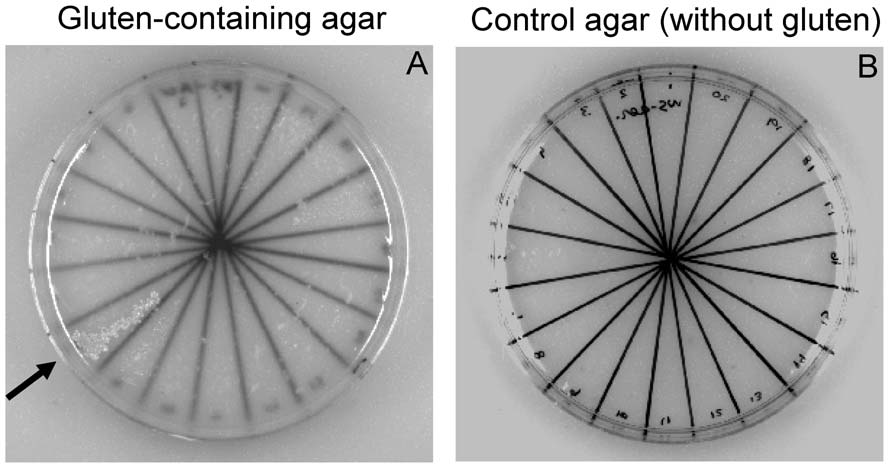
Assessment of bacterial growth on gluten-limited agar. Twenty different microbial strains collected from dental plaque were cultured on agar media containing gluten as the sole protein source (GA, left) and on the same agar formulation not containing gluten (control, right). After 24 h incubation, among these twenty analyzed strains, one strain (WSA-8) was found to be capable of growing on the gluten agar (indicated by an arrow). Note the presence of small, undissolved gluten particles appearing as white flakes in the gluten agar.

Oral microorganisms capable of degrading gluten were obtained by plating on gluten-limited agar plates (GA). Bacterial colonies were subcultured on Brucella agar to purity and further subcultured on GA to verify growth on this selective agar. Figure 1A shows an example of a segmented GA plate with 20 colonies subcultured from Brucella agar plates. One strain, indicated with an arrow, grew rapidly on GA. In control experiments, the strains were cultured on agar plates that contained all the ingredients of GA except wheat gluten (Figure 1B). The lack of growth on this agar formulation indicates that gluten is an essential ingredient facilitating growth. By this selective plating strategy, 27 aerobic and 30 anaerobic oral strains capable of metabolizing gluten were obtained. Microbial speciation of the fastest growing strains (5 aerobic and 10 anaerobic strains) was carried out by 16S rDNA analysis. The DNA typing results revealed that some of the strains represented the same genus. For instance, strains WSA-2B and WSA-26 were both typed as *Rothia mucilaginosa* ot 681. Strain WSA-8 also belonged to the *Rothia* genus, representing *Rothia aeria* ot 188 (formerly denoted as *Rothia sp.* ot 188; www.homd.org). Strain WSA-7A was identified as *Streptococcus mitis* ot 677 and WSA-10 as *Staphylococcus epidermis* ot 601. All anaerobic strains belonged to the *Bifidobacterium* genus: strains WSAN-14, -16, and -24 were typed as *Bifidobacterium longum* ATCC 15697; and strains PAN-5, -8, -18, and -19 were identified as *Bifidobacterium dentium* ot 588.

### Hydrolysis of paranitroanilide-derivatized substrates

The tripeptides YPQ, QQP, PPF and PFP occur with high frequency in gluten sequences. In our earlier work we have demonstrated that mixed human dental plaque bacteria cleave Z-YPQ-pNA, Z-QQP-pNA, Z-PPF-pNA and Z-PFP-pNA [22]. Here we investigated if the 15 oral strains selected on GA would cleave these pNA-derivatized substrates (Table 1). In the aerobic category, *R. mucilaginosa* and *R. aeria* efficiently hydrolyzed Z-YPQ-pNA. Some activity was observed for *Streptococcus mitis* whereas *Staphylococcus epidermis* was inactive. In contrast to Z-YPQ-pNA, the substrates Z-QQP, Z-PPF-pNA and Z-PFP-pNA were not cleaved. The anaerobic bifidobacteria were unable to cleave any of the four gliadin-derived substrates.

**Table 1:** Enzymatic characteristics of selected oral microorganisms cultured on gluten agar.

Aer/Anaer	Strain ID	GenBank Accession	Strain name^a^	Hydrolysis of gluten-based substrates^b^			
				YPQ	QQP	PPF	PFP
Aerobic	WSA-2B	JF895494	*Rothia mucilaginosa* HOT-681	+++	−	−	−
	WSA-7A	JF895495	*Streptococcus mitis* HOT-677	+/−	−	−	−
	WSA-8	JF895496	*Rothia aeria* HOT-188	+++	−	−	−
	WSA-10	JF895497	*Staphylococcus epidermidis* HOT-601	−	−	−	−
	WSA-26	JF895498	*Rothia mucilaginosa* HOT-681	+++	−	−	−
Anaerobic	WSAN-14	JF895499	*Bifidobacterium longum* HOT-B54	−	−	−	−
	WSAN-16	JF895500	*Bifidobacterium longum* HOT-B54	−	−	−	−
	WSAN-24	JF895501	*Bifidobacterium longum* HOT-B54	−	−	−	−
	WSAN-25	JF895502	*Veilonella atypica* HOT-524	−	−	−	−
	PAN-0	JF895503	*Streptococcus pneumoniae* HOT-734	−	−	−	−
	PAN-5	JF895504	*Bifidobacterium dentium* HOT-588	−	−	−	−
	PAN-8	JF895505	*Bifidobacterium dentium* HOT-588	−	−	−	−
	PAN-18	JF895506	*Bifidobacterium dentium* HOT-588	−	−	−	−
	PAN-19	JF895507	*Bifidobacterium dentium* HOT-588	−	−	−	−
	PAN-23	JF895508	*Bifidobacterium dentium* HOT-588	−	−	−	−
Aer+Anaer	Whole plaque		Mixture of bacteria	+++	+++	+++	+++

a Speciation was carried out by partial 16S rDNA gene sequencing. All sequences were greater than 99% similar reference sequences at the Human Oral Microbiome Database (www.homd.org).

b Substrates (final concentration 200 µM) were mixed with bacterial suspensions in saliva ion buffer (OD_620_ 1.2). Hydrolysis was measured spectrophotometrically at 405 nm after 24 h incubation.

**Figure 2:**
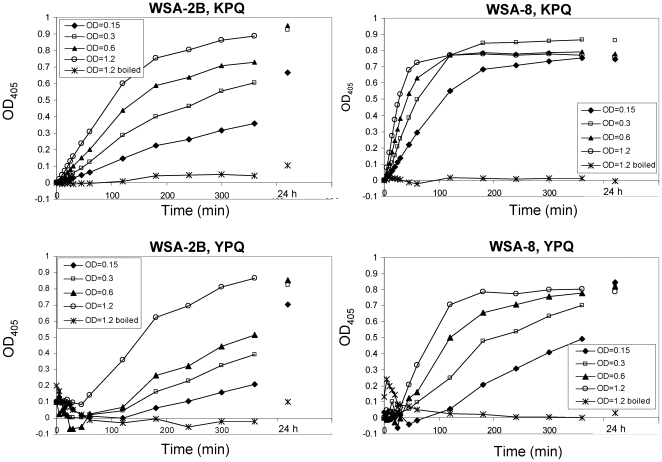
Relationship between cell density and proteolytic activity. WSA-2B (*R. mucilaginosa*) or WSA-8 (*R. aeria*) cells were suspended in saliva ion buffer to a final cell concentration of OD_620_ 0.15, 0.3, 0.6, and 1.2. Z-KPQ-pNA or Z-YPQ-pNA were added as enzymatic substrates to a final concentrations of 200 µM. Note that the rate of substrate hydrolysis increased with increasing cell density. As expected, boiled cell suspensions (OD_620_ 1.2) were devoid of enzyme activities.

For two of the aerobic strains, WSA-2B (*R. mucilaginosa*) and WSA-8 (*R. aeria*) cleavage kinetics were established towards Z-YPQ-pNA and another substrate of the XPQ type, namely Z-KPQ-pNA. Both substrates were hydrolyzed in a cell-density and time-dependent manner (Figure 2). At the highest cell densities evaluated (OD_620_ 1.2), *R. mucilaginosa* virtually completely hydrolyzed Z-YPQ-pNA and Z-KPQ-pNA after 360 min while *R. aeria* completed the cleavage of these substrates after 180 min and 60 min, respectively. Overall, the results reveal rapid, cell density dependent, enzyme kinetics for both *Rothia* strains. Cleavage of Z-YPQ-pNA as well as Z-KPQ-pNA indicates protease cleavage after PQ↓ and little influence of the amino acid in the P3 position on protease recognition.

### Gliadin degradation in solution

**Figure 3:**
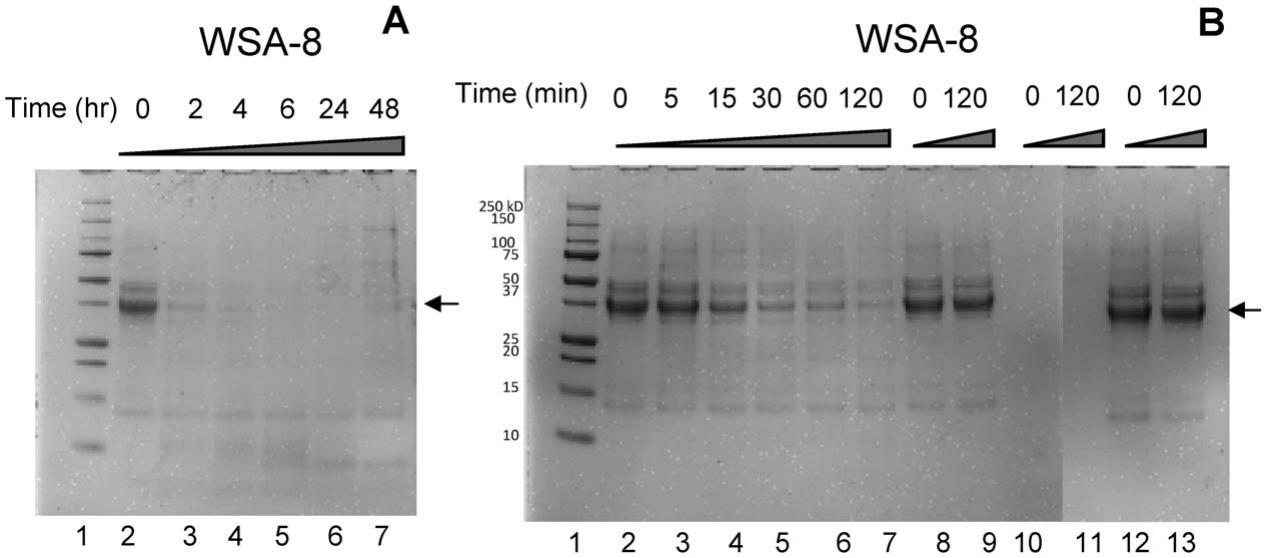
Degradation of gliadins by *R. aeria*. Gliadin (250 µg/ml) was incubated in a suspension of WSA-8 (*R. aeria*). The final OD_620_ was 1.0. Incubation sample aliquots were analyzed by Bis-Tris PAGE. A, lane 1: molecular weight standard; lanes 2–7, WSA-8/gliadin mixtures incubated for 0, 2, 4, 6, 24 and 48 h, respectively. B, lane 1: molecular weight standard, lanes 2–7: WSA-8/gliadin mixture incubated for 0, 5, 15, 30, 60 and 120 min, respectively. Lanes 8 and 9: gliadins incubated for 0 and 120 min in boiled bacterial cell suspensions; lanes 10 and 11: cell suspensions without added gliadins; Lanes 12 and 13: gliadins incubated for 0 and 120 min in saliva ion buffer only. Arrow points to the major protein constituent in the gliadin mixture.

The actual degradation of gliadin by oral bacteria was investigated by incubating gliadins in a suspension of strain WSA-8 (*R. aeria*). Aliquots were removed from the incubation mixture at the indicated time points and analyzed by SDS-PAGE (Figure 3). We previously reported that the major protein in the gliadin preparation, exhibiting a molecular weight of approximately 37 kDa, is degraded, although at a fairly low rate, in a suspension of mixed dental plaque [22]. Gliadins were however rapidly cleaved in a suspension of *R. aeria*, as evidenced from the finding that within 2 h of incubation the added amount of gliadin (250 µg in a 1 ml volume) was completely degraded (Figure 3A). The precise time course for gliadin degradation by *R. aeria* was established in a separate experiment in which sampling was carried out at shorter time intervals within the 2 h incubation time period (Figure 3B). Approximately 50% of the added gliadin amount was degraded in ∼30 minutes. Similar degradation kinetics were observed when gliadins were incubated in a suspension of strain WSA-2B (data not shown). These results demonstrate that *Rothia* species are highly effective in degrading gluten and that their activities far exceed the activities present in mixed dental plaque.

### Comparison of proteolytic degradation of the 33-mer and 26-mer by mammalian enzymes and by *R. aeria*

**Figure 4:**
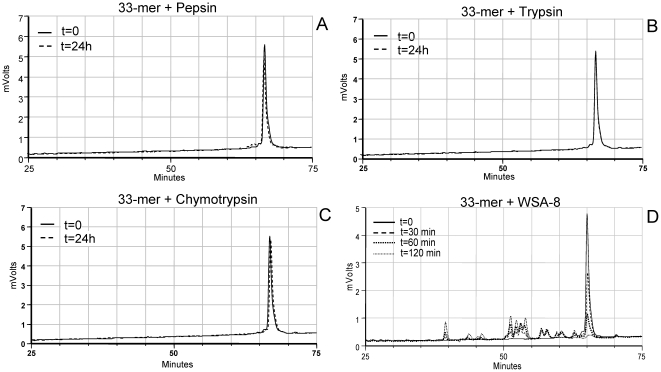
Degradation of the 33-mer by mammalian enzymes and by enzymes associated with *R. aeria*. The 33-mer peptide was incubated with pepsin (A), trypsin (B), chymotrypsin (C) (each 1 µg/ml) sampled at t = 0 and t = 24 h or in a suspension of WSA-8 (*R. aeria*) cells (D; OD_620_ 1.2) sampled at t = 0, 30 min, 60 min and 120 min. Degradation of the 33-mer in incubation aliquots was monitored by RP-HPLC.

The α-gliadin derived 33-mer peptide as well as the γ-gliadin derived 26-mer peptide are resistant to degradation by mammalian enzymes [6], [8] which we confirmed experimentally (Figure 4A, B, C). In parallel experiments, the peptides were incubated in a cell suspension of strain WSA-8 (*R. aeria*). Both the 33-mer and 26-mer peptide are rich in XPQ sequences, putatively targeted by the *Rothia* enzymes. Results show the rapid degradation of the 33-mer by *R. aeria* (Figure 4D) as evidenced from the disappearance of the 33-mer peptide over the 2 h time interval and the concomitant appearance of degradation fragments eluting earlier in the chromatogram. The 26-mer peptide was also sensitive to proteolytic degradation (Figure S1). The data indicate a dramatic difference in proteolytic capacity of mammalian digestive enzymes versus *Rothia* proteases towards the gliadin regions implicated in celiac disease.

### Determination of cleavage site specificity of *R. aeria* towards the 33-mer

**Figure 5:**
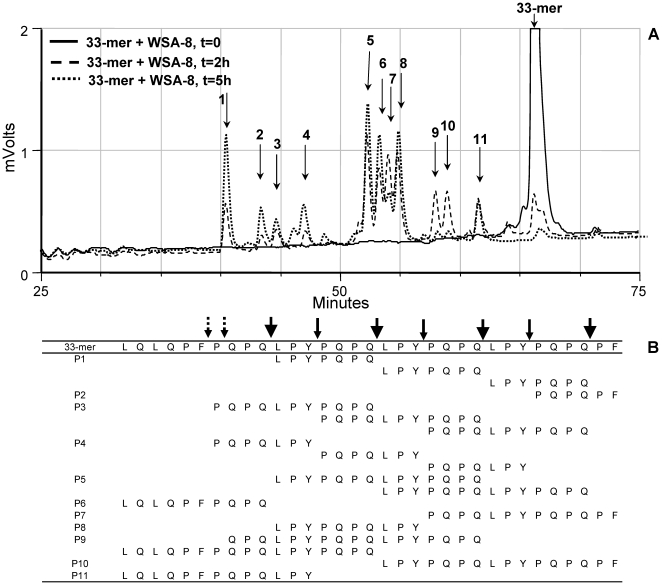
Degradation and fragment analysis of the 33-mer incubated with *R. aeria*. Gliadin 33-mer (250 µg/ml) was incubated in a suspension of WSA-8 (*R. aeria*) cells (OD_620_ 1.2). Incubation aliquots removed after 0 h, 2 h and 5 h were analyzed by RP-HPLC (A). Degradation peaks labeled 1 to 11 were collected and sequenced by LC-ESI-MS/MS (B). Large arrows: cleavage after QPQ; small solid arrows: cleavage after LPY; small dotted arrows: other cleavages.

Immunogenic epitopes contained within the 33-mer peptide include glia-α9 (LQLQPFPQPQLPY) and glia-α2 (PQPQLPYPQPQLPY), and in the 26-mer peptide glia-γ2 (PFPQQPQQP) and PYPQQPQQP [27]. It is of importance to determine if the *Rothia* enzymes could cleave in these particular domains. RP-HPLC analysis of the fragmentation cascade of the 33-mer in a cell suspension of WSA-8 (*R. aeria*) is shown in Figure 5A. The intact 33-mer eluted after 66 min (t = 0 sample, peak off scale). In the t = 2 h and 5 h samples the 33-mer was completely cleaved proteolytically. Degradation fragments in peaks 1 to 11 were collected and structurally characterized by LC-ESI-MS/MS analysis (Figure 5B). From the N- and C-termini of these peptides, the enzymatic cleavage site specificities of the *Rothia*-associated enzymes could be derived. Consistent with noted XPQ-pNA hydrolysis, prominent cleavage was observed after QPQ↓. Repeated cleavage was also noted after LPY. This novel protease specificity was confirmed with the synthetic enzymatic substrate Z-LPY-pNA (data not shown). Most degradation fragments had arisen through cleavage at QPQ↓ and/or LPY↓ with occasional cleavage at other positions. Some fragments in the 2 h sample (i.e. those eluting in peaks 9 and 10) disappeared in the 5 h sample, indicative of continued cleavage as time progressed. Degradation of the 33-mer was also monitored in a cell suspension of *R. mucilaginosa* ATCC 25296 (not shown). As for *R. aeria*, XPQ↓ and LPY↓ were the prominent protease target sites for *R. mucilaginosa*. Overall, *R. aeria* and *R. mucilaginosa* enzymes cleave the antigenic 33-mer peptide in domains that contain the major α-gliadin epitopes.

### Determination of cleavage site specificity of *R. aeria* towards the 26-mer

The proteolytic fragmentation cascade of the 26-mer by *WSA-8* (*R. aeria*)-associated proteases is shown in supplemental Figure S2. *R. aeria* as well as *R. mucilaginosa* cleaved the 26-mer peptide after XPQ as well as other glutamine (Q) residues along the 26-mer sequence. In addition, QPY was cleaved, possibly by the same enzyme(s) recognizing LPY in the 33-mer peptide. In analogy to observations made with the 33-mer peptide, it can be concluded that *Rothia* enzymes targeted regions in the 26-mer containing the essential γ-gliadin epitopes.

### Determination of enzyme molecular weights by gliadin zymography

**Figure 6:**
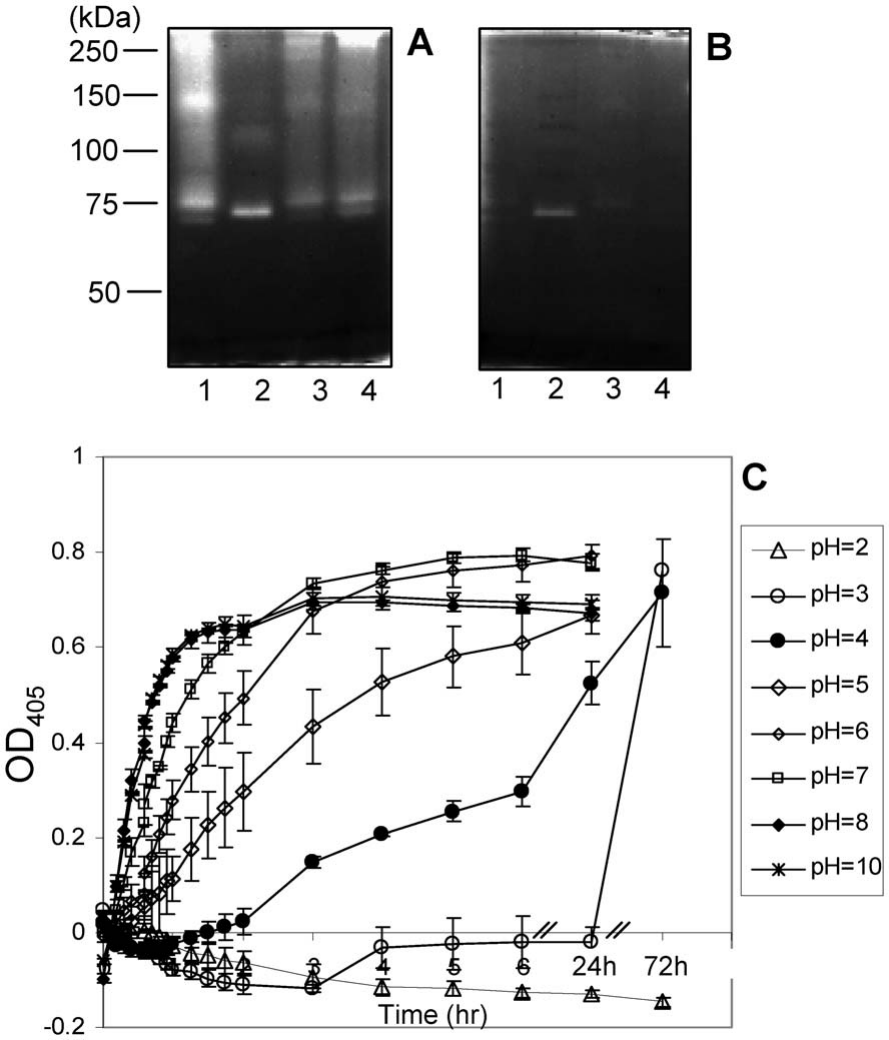
Gliadin zymography (6%) of *Rothia* strains and pH activity analysis. In the zymogram gels 150 µl cells (OD_620_ 5.0) were applied per lane. Lane 1, strain WSA-2B (*R. mucilaginosa*); lanes 2: strain WSA-8 (*R. aeria*), lane 3: strain WSA-26 (*R. mucilaginosa*); lane 4: strain ATCC 25296 (*R. mucilaginosa*). A, gel developed at pH 7.0; B, gel developed at pH 3.0. C, Z-YPQ-pNA (200 µM) hydrolysis by WSA-8 cells (OD_620_ 1.2) measured in 20 mM Tris solutions ranging in pH from 2.0 to 10.0. Measurements at 405 nm were carried out hourly for the first 6 hours and after 24 h and 72 h.

The next series of experiments were designed to gain more insight into structural characteristics of the gluten degrading enzymes. To establish the approximate molecular weight of the enzymes strains WSA-2B (*R. mucilaginosa*), WSA-8 (*R. aeria*) and WSA-26 (*R. mucilaginosa*) as well as reference strain, *R. mucilaginosa* ATCC 25296 were analyzed by gliadin zymography. All four strains expressed gliadin-degrading enzymes which appeared as clear bands in the zymogram gel developed at pH 7.0 (Figure 6A). As expected, the protease patterns of the *R. mucilaginosa* strains were very similar, showing a major double band in the 75 kDa region. Activity was also noted in higher (∼150 kDa) molecular weight regions, perhaps representing dimeric forms of the 75 kDa enzymes. *R. aeria* displayed one prominent protease band with an electrophoretic mobility around 70 kDa.

### pH activity analysis

To determine protease activities under conditions that are representative of the stomach, the zymogram gel with the four *Rothia* strains was also developed at pH 3.0 (Figure 6B). At this pH value the *R. mucilaginosa* enzymes were virtually inactive. In contrast, strain *R. aeria* showed a weak but distinct band with enzymatic activity in the zymogram. To further investigate the pH range over which strain *R. aeria* was active, Z-YPQ-pNA substrate hydrolysis was measured in solutions ranging in pH from 2.0 to 10.0. Optimal enzyme activities were observed at pH>7.0 (Figure 6C). Substrate hydrolysis rates decreased in parallel with decreasing pH values from pH 7.0 to pH 4.0. At pH 3.0 substrate cleavage was noted only after 72 h incubation. The slow but noticeable enzyme activity of *R. aeria* at this pH value is consistent with the gliadin zymogram results presented in Figure 6B. At pH 2.0, however, no Z-YPQ-pNA hydrolysis could be observed and protease activities could not be reactivated upon transfer to neutral pH values (data not shown), suggesting irreversible inactivation of the enzyme under these conditions.

## Discussion

The present study has uncovered specific microorganisms in mixed dental plaque that display potent gluten-degrading activity. The most efficient cleaving strains were identified as *Rothia mucilaginosa* and *Rothia aeria*. The data present evidence that even in the absence of gliadin pretreatment with mammalian digestive enzymes, gliadins (and by inference glutens) serve as a good substrate for *Rothia* associated bacterial enzymes and are rapidly cleaved. Importantly, major immunogenic epitopes that play a key role in celiac disease are also targeted by *Rothia* enzymes. The colonization of the oral cavity with microorganisms that produce glutamine endoproteases may not be surprising given that salivary proline-rich proteins as well as dietary wheat gluten proteins are prominent and abundant substrates in the oral cavity and an important factor in driving microbial colonization is nutrient substrate availability. The observations made suggest that *Rothia* bacteria may contribute to the digestive processing of immunogenic gluten proteins.

The human body is a major reservoir of a wide variety of microorganisms and their contribution to overall health and well-being of the host, including aspects of digestion, become more and more recognized [28]. The human microbiome project, a multidisciplinary international research initiative, is aimed at characterizing the microbial composition of the entire human body [29]. Among all human organ- and body-site specific microbiomes, the oral microbiome has been characterized most extensively (accessible at www.homd.org) [30]. The known microbiome of the oral cavity and its anatomically contiguous regions to date contains 619 taxa, derived from 13 phyla: Recently, an additional 36,043 gene clones were sequenced, identifying an additional 434 novel oral taxa which are candidates for addition to the database after validation [31]. Among all oral microbial clones sequenced to date, *R. mucilaginosa* ot 681 and *R. aeria* ot 188 rank at #170 and #221 in order of abundance. Evidently, the selective GA plating approach applied in our study has significantly enriched for the *Rothia* bacteria in the mixed oral samples.

*R. mucilaginosa* and *R. aeria* belong to the *Rothia* genus under the *Actinobacteria* phyla. *Rothia* species are Gram-positive bacteria, ovoid in shape and form clusters of cocci. *R. aeria*, named after its isolation from air in the Russian space laboratory Mir [32], is an oral colonizer [31]. *R. mucilaginosa* also primarily colonizes the oral cavity [33] but has furthermore been isolated from other body sites, including the upper respiratory tract and the duodenum [34], [35], [36]. Colonization of the duodenum with gluten-degrading *Rothia* species is of particular interest in view of the fact that mucosal damage in celiac disease is largely restricted to this region of the GI tract. Bacterial speciation of 2,247 clones recovered from 63 duodenal biopsies obtained from healthy and celiac patients showed that *R. mucilaginosa* comprised ∼6% of the clones and was present in ∼65% of the biopsies, identifying it as a true colonizer of the duodenum [36]. No significant differences were found in the number of *R. mucilaginosa* clones from healthy and celiac patients (6.5% and 5.9% of clones, respectively). Likewise, the percent of healthy and celiac biopsies being positive for *R. mucilaginosa* did not differ significantly from each other (64% and 67%, respectively) [36]. It thus appears that *R. mucilaginosa* colonizes the healthy and celiac-afflicted duodenum to the same extent. It will be of interest to also investigate *Rothia* concentration differences in human saliva in view of the fact that the microbial colonization levels in the oral cavity far exceed those of the duodenum, and a large volume of saliva is swallowed each day (approximately 0.8–1.0 L).

The discovery of salivary microorganisms degrading dietary proteins *in vitro* prompts the question to what extent such microorganisms play a role in food processing *in vivo*. During mastication (chewing) foods are mixed with whole saliva helping to accelerate the break-down by digestive enzymes during the residency time in the oral cavity. Oral microorganisms in the swallowed food bolus may or may not survive and/or continue to exert proteolytic activities during or after gastric passage. Our *in vitro* data with *R. aeria* show that its enzymes are not abolished at acidic pH values, and are optimally active under more basic pH conditions. *In vivo*, this could mean that during gastric passage the enzymes will neither be active nor destroyed, and that enzymatic reactivation would occur upon transfer to the duodenum. With regard to duodenal *Rothia* enzyme activity, it is relevant that *R. mucilaginosa* gains a foothold in the duodenum [36]. This offers the intriguing possibility that *Rothia* may colonize the duodenum and perform proteolytic activities locally in conjunction with mammalian-derived enzymes to degrade gluten.

From a therapeutical perspective, the identified *Rothia* enzymes differ in three major aspects from the major glutenase enzymes that are currently being tested in clinical trials (SC-PEP and EP-B2; Alvine Pharmaceuticals, and AN-PEP; DSM). First, the enzyme source is a resident human body-associated oral commensal bacterium. Second, the cleavage site specificity of the *Rothia* enzymes is unique in that they cleave XPQ↓ as well as LPY↓. The latter cleavage specificity is not exerted by prolyl endopeptidases, which hydrolyze peptide bonds C-terminal to proline residue [11], [12]), and is also not observed for EP-B2 [13]. Thus the *Rothia* specificities supplement those of enzymes under investigation for celiac disease therapy. Cleavage after the LPY↓ tripeptide is highly relevant since it is present in three copies throughout the immunogenic 33-mer sequence. The third feature that sets the *Rothia* enzymes apart is their wide pH activity range spanning from pH 4.0 (appreciable activity) to pH 10.0. While at this stage of our investigation we are unable to assign the activity to one or to several enzymes in the *Rothia* cell suspension, the potential of *Rothia* to serve as a valuable natural source of gluten-degrading enzymes is demonstrated.

With regard to the suggested *in vivo* usefulness of the *Rothia* enzymes as a treatment for celiac disease, it is imperative to establish if gliadin fragmentation by *Rothia* is beneficial or harmful to the host, in particular, to the celiac host. While immunogenic domains in the α-2, α-9 and γ-gliadin epitopes were cleaved by the *Rothia* enzymes, digestion of these regions by *Rothia* was incomplete as evidenced by the finding that by-products of the digestion still contained immunogenic peptide sequences such as QLQPFPQPQLPY [37], PFPQPQLPY and PQPQLPYPQ [7] (Figure 5B and Figure S2B). By virtue of their smaller size, these peptides may reach the lamina propria more easily than larger immunogenic fragments [38]. Furthermore, most of the degradation fragments identified are theoretically of sufficient length to bind to HLA-DQ2 after deamidation by tissue transglutaminase [39], an essential step in the pathway towards T-cell activiation [40]. To test this, we measured TG2-mediated cross-linking of peptides in the *Rothia*-33-mer and *Rothia*-26-mer degradation mixtures to monodansyl cadaverine as a measure for deamidation and T cell stimulatory potential. A surprisingly strong correlation was observed between 33-mer or 26-mer degradation and loss of TG crosslinking. These results indicated that the degradation fragments are not good substrates for deamidation by TG2, suggesting loss of immunotoxicity [41]. T-cell proliferation assays are planned to validate these observations.

Gluten-degrading microorganisms in the GI tract may play a hitherto unappreciated role in the digestion/detoxification of dietary gluten. They open promising new avenues in the search for novel therapies to neutralize the deleterious effects of gluten in patients with celiac disease. Furthermore, to exploit these bacteria or their enzymes is highly attractive, since they belong to the normal flora of the upper GI tract.

## Supporting Information

Figure S1 Degradation of the 26-mer by mammalian enzymes and by enzymes associated with *R. aeria*. The 26-mer peptide was incubated with pepsin (A), trypsin (B), chymotrypsin (C) (each 1 µg/ml) sampled at t = 0 and t = 24 h or in a suspension of WSA-8 (*R. aeria*) cells (D; OD_620_ 1.2) sampled at t = 0, 30 min, 60 min and 120 min. Degradation of the 26-mer in incubation aliquots was monitored by RP-HPLC.
(TIFF)

Figure S2 Degradation and fragment analysis of the 26-mer incubated with *R. aeria*. Gliadin 26-mer (250 µg/ml) was incubation in a suspension of WSA-8 (*R. aeria*) cells (OD_620_ 1.2). Incubation aliquots removed after 0 h, 2 h and 5 h were analyzed by RP-HPLC (A). Degradation peaks labeled 1 to 10 were collected and sequenced by LC-ESI-MS/MS (B). Large solid arrows: cleavage after XPQ; large dotted arrows: cleavage after Q (except XPQ), small solid arrows: cleavage after QPY; small dotted arrows: other cleavages.
(TIFF)
