# Supplementary figures and images for: Identification of Rothia Bacteria as Gluten-Degrading Natural Colonizers of the Upper Gastro-Intestinal Tract
Source: PLoS One. 2011 Sep 21;6(9):e24455. doi: 10.1371/journal.pone.0024455 (PMC3177827; doi:10.1371/journal.pone.0024455)

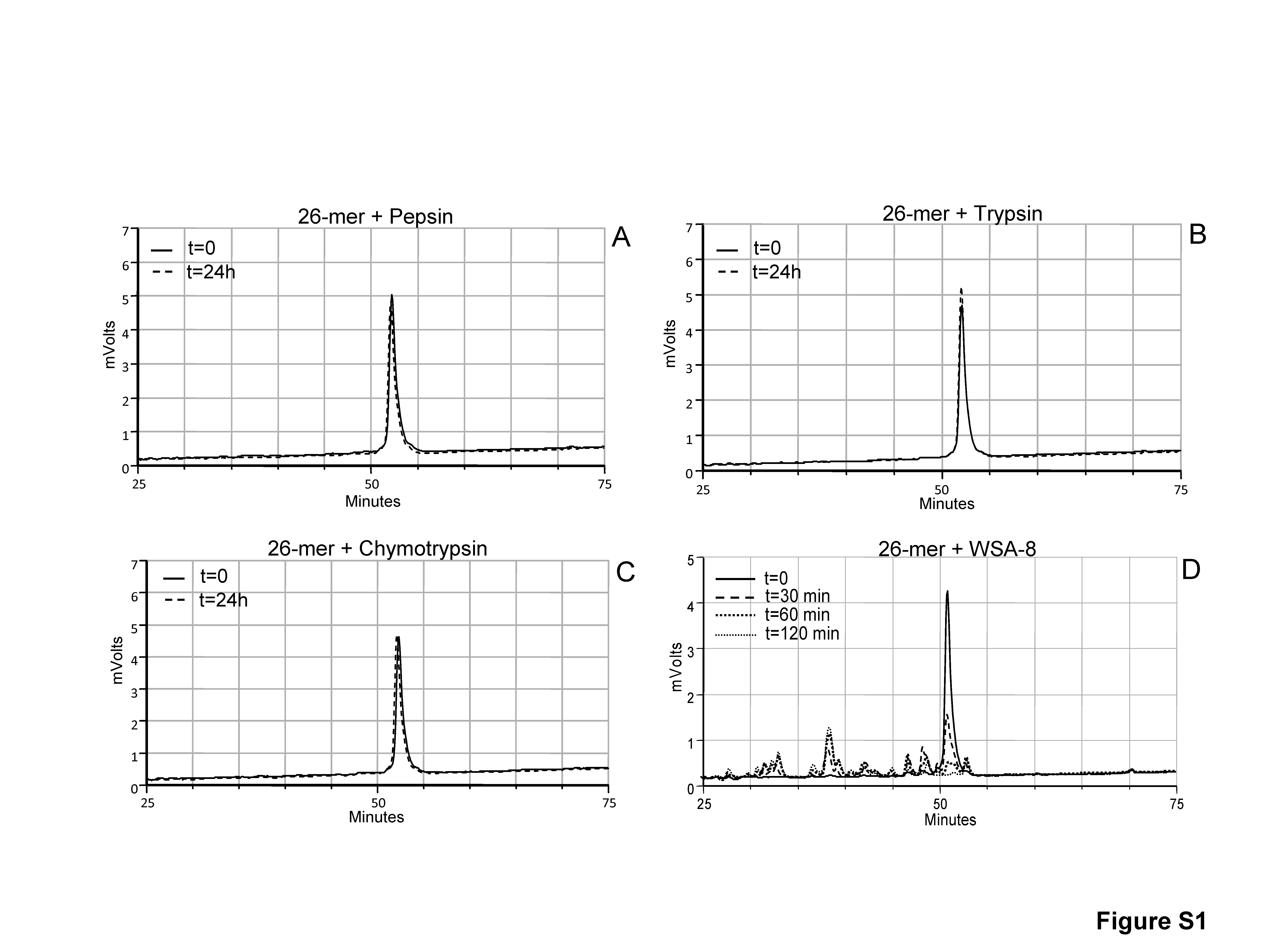

Supplement: Figure S1 — Degradation of the 26-mer by mammalian enzymes and by enzymes associated with R. aeria. The 26-mer peptide was incubated with pepsin (A), trypsin (B), chymotrypsin (C) (each 1 µg/ml) sampled at t = 0 and t = 24 h or in a suspension of WSA-8 (R. aeria) cells (D; OD620 1.2) sampled at t = 0, 30 min, 60 min and 120 min. Degradation of the 26-mer in incubation aliquots was monitored by RP-HPLC. (TIFF) [file pone.0024455.s001.tiff]

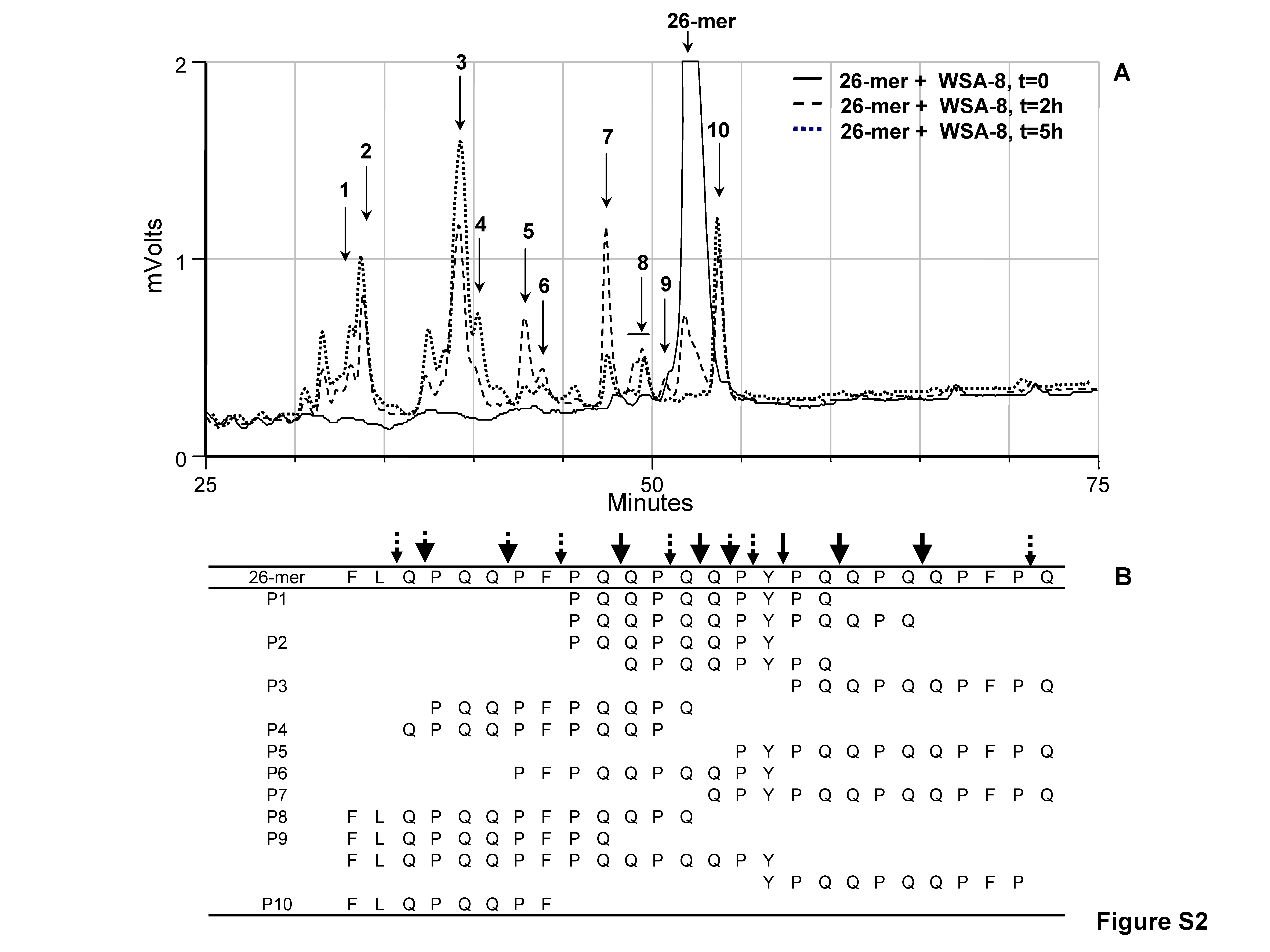

Supplement: Figure S2 — Degradation and fragment analysis of the 26-mer incubated with R. aeria. Gliadin 26-mer (250 µg/ml) was incubation in a suspension of WSA-8 (R. aeria) cells (OD620 1.2). Incubation aliquots removed after 0 h, 2 h and 5 h were analyzed by RP-HPLC (A). Degradation peaks labeled 1 to 10 were collected and sequenced by LC-ESI-MS/MS (B). Large solid arrows: cleavage after XPQ; large dotted arrows: cleavage after Q (except XPQ), small solid arrows: cleavage after QPY; small dotted arrows: other cleavages. (TIFF) [file pone.0024455.s002.tiff]
